# The Evolving Role of Intra-arterial Chemotherapy in Adult and Pediatric Cancers: A Comprehensive Review

**DOI:** 10.7759/cureus.46631

**Published:** 2023-10-07

**Authors:** Adarsh Vardhan Tangella

**Affiliations:** 1 Internal Medicine, Andhra Medical College, Visakhapatnam, IND; 2 Internal Medicine, King George Hospital, Visakhapatnam, IND

**Keywords:** adult cancer, pediatric cancer, hepatic malignancy, retinoblastoma, chemotherapy, primary intra-arterial chemotherapy, concomitant chemo-radiotherapy

## Abstract

The development of intra-arterial chemotherapy (IAC) was driven by an ambition to mitigate systemic side effects, enhance the bioavailability of drugs, and optimize the efficacy of chemotherapeutic agents. While the initial research on IAC primarily examined its effectiveness in treating various liver malignancies, the application of this treatment has undergone significant advancements since its introduction. The primary objective of this article is to examine the current range of utilization of IAC, both with and without radiotherapy, while also evaluating the results of relevant clinical trials. Furthermore, this article explores potential future advancements and opportunities in this field. From the scoping review of available articles, it can be concluded that IAC is an effective treatment alternative and, sometimes, a better first-line option, but there is a need for more evidence to make IAC a regular treatment option available for patients.

## Introduction and background

Intra-arterial chemotherapy (IAC), also referred to as intra-arterial infusion therapy, involves directly administering chemotherapy drugs into the arteries responsible for supplying blood to a particular organ or tumor. This approach aims to optimize the localized concentration of chemotherapy drugs within the intended region while concurrently minimizing their systemic exposure, thereby mitigating adverse effects [1]. During the 1950s and 1960s, medical professionals initiated investigations into the concept of administering chemotherapy medications directly to the specific location of the tumor or the affected organ to augment the effectiveness of treatment and mitigate adverse reactions [2]. Consequently, the emergence of methodologies such as IAC ensued. Over the course of time, there has been a notable advancement in the sophistication of techniques employed in the administration of IAC [3]. The advancement of angiography and radiologic imaging techniques has played a crucial role in facilitating the precise positioning of catheters within the desired arteries, thereby ensuring the accurate administration of drugs [4]. The advancement of novel chemotherapy medications and the exploration of drug combinations have significantly enhanced the efficacy of treatment interventions [5]. Intra-arterial chemotherapy is frequently employed in conjunction with various therapeutic modalities, including surgical intervention, radiation therapy, and targeted therapy, to establish a comprehensive and multifaceted strategy for managing cancer [6-10].

Scope of usage of IAC

**Figure 1:**
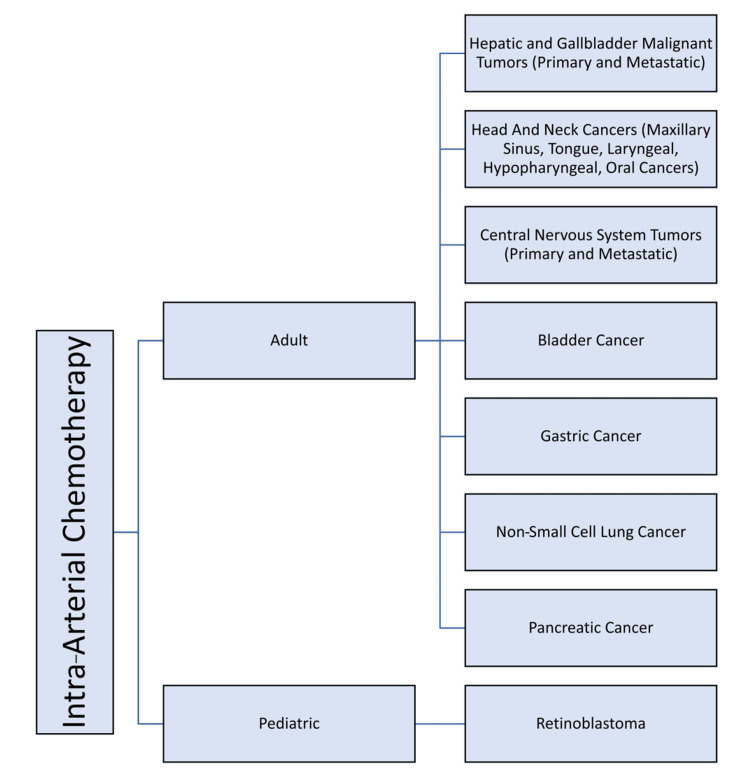
Spectrum of usage of intra-arterial chemotherapy in pediatric and adult populations

As previously mentioned, hepatic malignant lesions were among the first tumors to be subjected to IAC. Currently, the utilization of IAC in the pediatric demographic is predominantly employed to manage retinoblastoma [1]. In the adult population, IAC has been employed as a therapeutic approach for a range of cancer types, encompassing primary and metastatic hepatic malignancies, bladder cancer, head and neck cancers, non-small cell lung cancer, pancreatic cancer, cervical cancer, as well as primary and metastatic central nervous system tumors [1]. This article is based on a review of relevant studies conducted on the utilization of IAC from 2010 to 2023. Figure 1 shows a flowchart depicting the present spectrum of usage of IAC.

## Review

Retinoblastoma

One of the earliest studies on IAC in retinoblastoma was done by Abramson et al. [11], wherein 28 eyes of 23 newly diagnosed retinoblastoma patients were examined, out of which 25 were Reese Ellsworth Grade (RE)-V. The route chosen for these patients was via the ophthalmic artery. Twelve patients were given melphalan; seven were given a combination of melphalan and topotecan; three were treated with a combination of melphalan, topotecan, and carboplatin; and one was treated with a combination of melphalan and carboplatin. The three-year survival rate was 100%, and only one out of the 28 eyes treated had to be enucleated due to progression. The Kaplan-Meier curve denoting enucleation-free survival was 100% at 12 months and 89% at two years [11]. This extremely positive outcome established the definitive role of IAC in high-grade retinoblastoma. Another prospective registry by Gobin et al. over four years of treating 95 eyes of 75 patients using selective catheterization of the ophthalmic artery and melphalan with or without topotecan-based chemotherapy showed that the ocular event-free survival rates at two years were 70%, out of which the event-free survival of eyes that received IAC as primary treatment was 81.7% and 58.4% for eyes that had previous treatment failure with intravenous chemotherapy and/or external beam radiotherapy [12]. In a study by Parareda et al., 12 eyes (eight primarily treated with IAC) of 11 patients were prospectively studied for outcomes using melphalan IAC every 21 days. With a median follow-up time of 29.5 months, ocular salvage was obtained in seven out of 12 eyes (58%). Both eyes were preserved with no tumor activity 56 and 30 months after the last IAC melphalan therapy [13].

Min Hahn et al. pointed out that exclusive IAC cannot exclude the risk of occult micrometastases in the central nervous system in advanced-stage retinoblastoma. Hence, they adopted an alternative intravenous (IVC) and IAC regimen wherein 13 eyes of 12 patients (10/13 eyes were Groups D or E International Classification for Retinoblastoma (ICRB)) were retrospectively studied for a median follow-up period of 30.4 months, and the overall salvage rate was 63.9% +/- 14.7% [14]. In an attempt to compare IVC and IAC regimens, Chen et al. performed a study [15] that proved that the ICRB classification-based overall success rate of IAC is higher than IVC (75.7% vs. 69.5%), and the global salvage rate with IAC was significantly higher in Group D ICRB (79.5% vs. 55.1%) but not in Group B (95.8% vs. 82.5%, P = 0.163) or Group E (51.2% vs. 43.2%, P = 0.578). There was no significant difference between IAC and IVC regarding recurrence or metastasis rates (15.0% vs. 15.4%, P=0.148, and 2.7% vs. 0.6%, P=0.194). For RE grading, IAC had a higher global salvage in groups IV (90.9% vs. 66.3%, P=0.047) and V (83.2% vs. 59.9%, P=0.003) but not in groups I-III. The overall success rate was higher in IAC than in IVC (87.1% vs. 77.3%, P=0.033) [15]. In a meta-analysis performed by Ravindran et al., the outcomes in a total of 873 patients and 1467 eyes were studied. 11.8% (174/1467) eyes were enucleated, metastatic disease occurred in 8/513 patients (1.6%), and global salvage was achieved in 318/906 (35.6%) cases of advanced retinoblastoma [16]. These studies have shown that melphalan-based IAC has been adopted as the current standard of care for advanced retinoblastoma (ICRB Groups D and E).

Hepatic malignant lesions

Oxaliplatin-Based Hepatic Arterial Infusion (HAI) in Advanced Colorectal Cancer With Hepatic Metastases

Dealing with hepatic metastases has drastically evolved, from systemic chemotherapy to trans-arterial chemoembolization. The earliest study, as per the period we have selected, comes from Tsimberidou et al., in which a phase 1 trial was done to evaluate the efficacy of hepatic arterial infusion of escalating doses of oxaliplatin along with systemic chemotherapy with 5-fluorouracil (5-FU), leucovorin, and bevacizumab. Fifty-seven patients were treated; the most common cancer was colorectal (n=27). Out of the 55 patients available for response evaluation, four (7%) had a partial response (PR), and 32 (58%) had stable disease (SD), out of which 15 had an SD >= four months. Of the 28 colorectal cancer patients, three (11%) had a PR, and nine (32%) had an SD for >= four months [17].

Goere et al. performed a retrospective study [18] where the role of oxaliplatin HAI with systemic 5-FU and leucovorin was studied in patients with initially unresectable colorectal liver metastases with or without a history of failure of systemic chemotherapy followed by radical surgery. The outcomes of a total of 87 patients were analyzed, and 69 patients had a history of previous failed systemic chemotherapy. Eighty-six percent of the patients had massive unresectable liver involvement. Catheter-related problems were observed in 31 patients but only led to the stoppage of HAI in 7. A total of 23 patients were operated on. The five-year overall survival (OS) was 56% in the surgery group versus none in the non-surgery group (p<0.0001). Ten out of 23 operated patients had a recurrence at a median follow-up of 63 months. This proved that the unresectability of metastatic liver masses can be dealt with successfully by using a combination of oxaliplatin HAI and systemic 5-FU with leucovorin [18].

Boileve et al. conducted a retrospective analysis to understand the role of oxaliplatin HAI in combination with systemic chemotherapy and targeted therapy (cetuximab, panitumumab, or bevacizumab) in unresectable colorectal liver metastases. Eighty-nine patients with a history of a median of one systemic chemotherapy regimen were included. Sixty-three percent received LV5FU2 (leucovorin + 5-FU), 36% received FOLFIRI (5-FU+leucovorin+irinotecan), 30% received cetuximab/panitumumab, and 70% had a history of bevacizumab-based regimens. Forty-five percent of the patients had stable disease, and 27% could undergo complete resection or ablation of colorectal liver metastases. The median OS and progression-free survival were 20 and nine months, respectively, at a median follow-up of 72 months. This showed that oxaliplatin HAI and systemic chemotherapy can be a promising, safe solution after initial chemotherapy failure [19].

Irinotecan-Based HAI in Advanced Colorectal Cancer With Hepatic Metastases

Eichler et al. performed a study to understand the role of irinotecan-based HAI in patients with advanced colorectal cancer with hepatic metastases. Imaging follow-up using Response Evaluation Criteria in Solid Tumors (RECIST) criteria showed a complete response in one patient, SD in 31 patients, and progressive disease in one patient. The median time for progression was 4.7 months, and the median overall survival was 15.6 months [20].

Irinotecan-Loaded Drug-Eluting Beads (DEBIRI)-Based Hepatic Artery Infusion in Advanced Colorectal Cancer With Hepatic Metastases

A study was done by Fiorentini et al. to evaluate the role of irinotecan-loaded DEBIRI in managing hepatic metastases from colorectal cancer [21]. Seventy-four patients were randomly assigned to receive DEBIRI (n=36) vs. systemic irinotecan, fluorouracil, and leucovorin (FOLFIRI, n=38). At 50 months, the OS was significantly longer for patients treated with DEBIRI over FOLFIRI (P=0.031). The median survival was 22 months for DEBIRI and nine for FOLFIRI. Progression-free survival was seven months for DEBIRI and four months for FOLFIRI, with the DEBIRI arm being significantly more extended (P=0.006). Extrahepatic progression was seen in all patients but took 13 months in the DEBIRI group vs. nine months in FOLFIRI, although the difference was not statistically significant. A crucial observation in this study was the statistically significant longer duration taken for patients in the DEBIRI group to show any improvement in quality of life (eight months vs. three months in FOLFIRI, P=0.00002) [21].

Building on the idea of DEBIRI as described earlier, a study by Pernot et al. aimed to investigate the role of intra-arterial DEBIRI along with a modified 5-FU, leucovorin, and oxaliplatin (mFOLFOX6) regimen in unresectable liver metastases from colorectal cancer. Of the 57 patients participating in this single-arm phase 2 study, nine-month progression-free survival (PFS) was 53.6%. The objective response rate based on RECIST criteria was 73.2%. The median follow-up was 38.3 months, the median OS was 37.4 months, and the median PFS was 10.8 months. However, the conclusion was not to recommend front-line DEBIRI + mFOLFOX6 due to the inability to meet the hypothesized nine-month PFS, high response rate, deep responses, and prolonged OS point out toward the future scope of integrating biologic agents, especially in patients with secondary surgery, amongst their goals [22].

Anti-Carcinoembryonic-Antigen (CEA) Chimeric Antigen Receptor T-cells (CAR-T)-Based Hepatic Artery Infusion in Advanced Colorectal Cancer With Hepatic Metastases

Katz et al. took the HAI studies to the immunological level by conducting a phase I trial of HAI of CAR-T against CEA-expressing adenocarcinomas metastatic to the liver [23]. The anti-CEA CAR-T cells were given to a total of nine patients, out of which six received the dose escalation regimen to understand the maximum tolerated dose (MTD), and three were given the maximum planned dose along with systemic interleukin-2 (IL-2) support. All of them had advanced disease and received a mean of 2.5 lines of conventional systemic therapy before enrollment. Four patients had more than 10 liver metastases. One patient was alive with SD at 23 months following anti-CEA CAR-T, and five died of progressive disease. In the group that received systemic IL-2 support, the CEA levels were reduced by 37% from baseline. This demonstrated the safety of anti-CEA CAR-T cells for managing advanced metastases [23].

Building on this idea, the HITM-SURE trial was performed, which evaluated the role of hepatic immunotherapy for metastases in a Phase 1b trial using anti-CEA CAR-T-based HAI utilizing a pressure-enabled drug delivery system (PEDD). The PEDD system helped overcome the challenge of reduced CAR-T cell penetrability in solid tumors. Interleukin-2 was given as a continuous intravenous infusion to support CAR-T in vivo. After the treatment, a PET-CT showed a complete metabolic response within the liver, which was durable and sustained for 13 months. This response was also accompanied by the normalization of serum tumor markers and an abundance of CAR+ cells in the post-treatment tumor specimens [24].

5-Fluorouracil-Based HAI in Advanced Colorectal Cancer With Hepatic Metastases

Kusano et al. performed a phase III trial to compare HAI with systemic chemotherapy after curative resection for liver metastasis of colorectal cancer. Ninety-one patients were randomized to receive 5-FU via continuous venous infusion (CVI) or intrahepatic arterial weekly high-dose 5-FU (WHF). The five-year OS was 59% in the CVI arm and 35.9% in the WHF. They concluded that HAI therapy has a certain protective effect against metastases after curative resection but does not significantly improve OS [25]. In a retrospective study done by Sato et al., a total of 137 patients who were refractory to systemic chemotherapy, including three cytotoxic agents (fluoropyrimidine, oxaliplatin, and irinotecan) with two molecular-targeted agents (bevacizumab and epidermal growth factor receptor antibody (cetuximab or panitumumab)), were given continuous infusion HAI with 5-FU for unresectable liver metastases. The median OS was 4.8 months, with an objective overall response rate and disease control rate of 12.4% and 64%, respectively. This showed that HAI with 5-FU can be an optional last-line chemotherapy [26].

A systematic review and meta-analysis performed by Buisman et al. tried to understand the role of adjuvant HAI chemotherapy (HAIC) in patients with resected colorectal liver metastases. The HAIC was associated with an improved OS (pooled hazard ratio (HR) 0.77; 95% CI 0.62-0.94). The survival benefit was more pronounced in studies using floxuridine (metabolically activated into 5-FU) (HR 0.76; 95% CI: 0.62-0.94), using a subcutaneous pump (HR 0.71; 95% CI: 0.61-0.84), and concomitant adjuvant systemic chemotherapy (HR 0.75; 95% CI: 0.59-0.96). This showed that adjuvant HAIC, especially with floxuridine or 5-FU in patients with resectable colorectal hepatic metastases, is a promising approach with good outcomes [27].

Comparison Between Different Interventional Therapies Available for Management of Unresectable Colorectal Cancer Liver Metastases

A meta-analysis done by Zhao et al. tried to evaluate the efficacy of HAI, conventional transarterial chemoembolization (TACE), drug-eluting embolic TACE (DEE-TACE), transarterial radioembolization (TARE), and their combinations with systemic chemotherapy (SCT). Amongst patients treated first line, DEE-TACE + SCT had the best survival outcomes, with a median OS of 26.5 months and a three-year restricted mean survival time (RMST) of 23.6 months. The DEE+TACE+SCT also had the highest pooled survival rate of 81.9% at one year and 66.1% at two years. The DEE+TACE+SCT and HAI+SCT had the highest pooled response rate of 56.7%, making HAI the next best therapeutic technique after DEE+TACE+SCT [28].

Bevacizumab-Based HAI in Advanced Colorectal Cancer With Hepatic Metastases

In a recent study done by Rigault et al., 10 patients were treated with HAI of bevacizumab along with systemic capecitabine and oxaliplatin or irinotecan. The patients included had dominant or unresectable colorectal liver metastases. They received a median of six cycles. A partial response was seen in two patients (20%). All patients died of disease progression. The median OS and progression-free survival were 14 months and 5.4 months, respectively. No deaths occurred due to drug toxicity. Although the study had to be halted due to poor funding and fewer patients, it did show that bevacizumab HAI can be safely used alongside systemic chemotherapy in patients with unresectable colorectal liver metastases [29].

Advanced Uveal Melanoma with Hepatic Metastases

In a multicentric randomized trial to evaluate the role of HAI versus systemic (IV) fotemustine in the management of advanced uveal melanoma limited to liver metastases, Leyvraz et al. randomized 171 patients. The HAI arm did not significantly improve OS compared to the IV arm (14.6 months vs. 13.8 months; HR: 1.09; CI 95%: 0.79-1.50). However, a significant PFS was observed in the HAI arm compared to the IV arm (4.5 months vs. 3.5 months, HR 0.62; 95% CI 0.45-0.84; p=0.002). The one-year PFS rate was 24% in the HIA arm versus 8% in the IV arm. The HAI arm also observed an improved response rate (RR) (10.5% vs. 2.4%). This showed that HAI with fotemustine can have a beneficial effect on PFS and RR but does not have any significant impact on OS [30].

Hepatocellular Carcinoma (HCC)

Brandi et al.’s Phase I study on continuous irinotecan HAI in patients with locally advanced HCC showed initial safety and efficacy for using a 25 mg/m(2)/day dose of irinotecan, and they reported a median time-to-progression of 11.3 months [31]. In a study done by Kasai et al., the efficacy of 5-FU and pegylated interferon (PEG-IFN) alpha-2b in patients with advanced HCC was studied. Fifty-nine patients with advanced HCC complicated by portal vein tumor thrombosis were given HAI 5-FU and subcutaneous PEG-IFN alpha-2b. The early response rate was 73%. Cumulative progression-free survival rates were 67.4% at six months, 30.2% at 12 months, 25.9% at 18 months, and 20.7% at 24 months. Cumulative survival rates were 82.4% at six months, 73.6% at 12 months, 52.8% at 24 months, and 44% at 36 months. The median survival time reported was 29.9%. Although there is a need to evaluate the survival benefit by conducting a large-scale trial, these results show that a combination of HAI 5-FU and PEG-IFN alpha-2b can be an effective solution in cases of advanced HCC [32].

Okusuka et al. ran a miriplatin (SM-1355) trial to evaluate its role in HAI for hepatocellular carcinoma. A total of 122 patients were evaluated for efficacy and toxicity. The two-year and three-year survival rates were 75.9% and 58.4%, respectively, and the adverse effects were mild. This showed that miriplatin can be safely used in cases of HCC and might be helpful [33]. In a Phase II trial by Ikeda et al., the efficacy of continuous HAI of 5-FU, mitoxantrone, and cisplatin for advanced hepatocellular carcinoma was studied, and, with an overall median survival time of 11.3 months, a one-year survival rate of 46.9%, and a median progression-free survival of seven months, 5-FU, mitoxantrone, and cisplatin HAI were concluded to be a safe and efficacious option in patients with advanced HCC [34].

Significant progress in HAI therapy for massive unresectable HCC came from the results of a prospective, non-randomized study by He et al. They compared the outcomes of TACE and HAI chemotherapy with the mFOLFOX regimen. In the mFOLFOX HAI arm, patients received oxaliplatin HAI, leucovorin HAI, a 5-FU bolus, and a continuous infusion. In the TACE arm, epirubicin, lobaplatin, mitomycin, lipiodol, and polyvinyl alcohol particles were used. The HAI chemotherapy arm had significantly superior partial response and disease control rates than TACE (52.6% vs. 9.8%, p<0.001; 83.8% vs. 52.5%, p<0.004). The median time to progression for HAI and TACE arms was 5.87 and 3.6 months, respectively. The resection rate was higher in the HAI arm (10 vs. 3, p=0.033). This proved that HAI with oxaliplatin is a better treatment alternative than TACE in cases of massive unresectable HCC with higher response rates and resection rates [35].

In a phase I trial, Guiu et al. evaluated the role of HAI idarubicin infusion in unresectable HCC. Out of the 15 patients who were enrolled, an objective response rate of 29% was noted after the second session of infusion. A median time to progression of 5.4 months and a median overall survival rate of 20.6 months were observed. This proved that two sessions of HAI with idarubicin are safe and efficacious in patients with unresectable HCC [36]. In summary, HAI infusions with oxaliplatin have shown some of the best outcomes in locally aggressive and massively unresectable HCC patients and can be considered an efficacious line of treatment.

Gastric cancer

In a retrospective study performed by Li et al. to understand the role of intravenous and intra-arterial intensified neoadjuvant chemotherapy in patients with advanced gastric cancer, a total of 56 patients with stage II or higher disease received intravenous 5-FU, intravenous leucovorin, and IAF of etoposide and cisplatin. After two cycles of preoperative chemotherapy, patients with resectable tumors underwent surgery. Of the 56 patients, the overall response rate was 78.57%, with 7.14% (4/56) reaching a complete clinical response. The R0 resection rates for prechemotherapy-resectable and unresectable diseases were 96.15% and 66.67%, respectively. A pathologically complete response was seen in 8.70% of the patients. The five-year survival rates for the whole and initially resectable groups were 21.8% and 42.3%, respectively. The median survival for initially resectable patients was 41 months, and for initially unresectable patients, it was 18 months. This shows that a combined intensified regimen is a feasible option as a pre-operative measure to improve resection and complete response rates [37].

The role of the 5-FU+ leucovorin + etoposide + oxaliplatin + epirubicin (FLEEOX) regimen in the management of unresectable locally advanced gastric cancer was analyzed by He et al., who administered 5-FU and leucovorin intravenously and etoposide and oxaliplatin intra-arterially using the Seldinger method in 105 patients. The response rate of preoperative chemotherapy was 78.1%, with a complete response in seven patients and a partial response in 75 cases. Seventy-eight patients could undergo surgery, out of which 67 achieved R0 resection. The one-year and three-year overall survival rates of all 105 patients were 71.9% and 31.7%, respectively, with a median survival time of 18 months. The one-year and three-year overall survival rates amongst the 78 patients who received chemotherapy and underwent surgery were 84.5% and 40%, respectively, with a median survival time of 30 months. Patients who underwent a preoperative chemotherapy infusion followed by surgery had a significantly longer overall survival time than those who underwent only chemotherapy (p<0.01) [38]. These studies have shown that IAC benefits patients with gastric cancer, especially unresectable or advanced gastric cancers, by making them more amenable to surgery and improving overall survival rates.

Cervical cancer

The role of IAC has been primarily studied in patients with bulky stage IB to stage IIIB cervical cancer by administering it via the internal iliac artery. These studies have focused on the role of avoiding postoperative irradiation and making the tumor resectable. In a study performed by Ujihara et al., the role of neoadjuvant intra-arterial chemotherapy (NAIC) to avoid radiation therapy was analyzed. Fifty-two patients received NAIC with combined cisplatin, epirubicin, mitomycin-C, and 5-FU. They were scheduled for a radical hysterectomy after two cycles. Out of the 52 patients, 51 underwent surgery. The overall positive response to NAIC was 88.5%. The three-year relapse-free survival rate and overall survival rates were 80.5% and 77.8%, respectively. Ten out of 52 patients had a recurrence. Seven out of 52 patients died from the disease during follow-up. Two prognostic factors- node status (positive vs. negative) and histological effect of NAIC-were significantly associated with disease prognosis (p=0.024 and p=0.021, respectively) [39]. These findings are supported by a prior study by Yamakawa et al., where a retrospective analysis of the outcomes of 26 patients who received NAIC was analyzed. The NAIC regimen included bleomycin, mitomycin-C, and cisplatin. The incidence of lymph node metastasis, parametrial infiltration, and vascular space involvement in 15 patients who received NAIC followed by radical surgery was significantly lower than the control group (13.3, 6.7, and 13.3% vs. 54.2, 43.8, and 60.4%). The five-year survival rate was significantly higher for all 26 patients who received NAIC (80.0%) compared to the control group (59.6%). Stage II and III cervical cancer patients who received NAIC had a significantly higher five-year survival rate when compared to the control groups [40].

Pancreatic cancer

A systematic review and meta-analysis by Liu et al. compared the outcomes of regional intra-arterial chemotherapy (RIAC) versus systemic chemotherapy (SC) in patients with Stage III/IV pancreatic cancer. The most commonly used agents were gemcitabine and cisplatin. Out of 298 patients, eight achieved complete remission with RIAC, whereas none achieved complete remission with SC. Patients who received RAIC had a better partial response (RR = 1.99, 95% CI: 1.50, 2.65; 58.06% with RIAC and 29.37% with systemic treatment), superior clinical benefits (RR = 2.34, 95% CI: 1.84, 2.97; 78.06% with RIAC and 29.37% with systemic treatment), complication rates (RR = 0.72, 95% CI: 0.60, 0.87; 49.03% with RIAC and 71.33% with systemic treatment), and side effect profiles (RR = 0.76, 95% CI: 0.63, 0.91; 60.87% with RIAC and 85.71% with systemic treatment). Patients who received RIAC had a longer median survival time of five to 21 months than those who received SC (2.7-14 months). The one-year survival rates were also higher in the RIAC group (28.6%-41.2%) compared to the SC group (0%-12.9%). These findings showed that RIAC is more efficacious and has much better outcomes when compared to systemic chemotherapy in the management of stage III/IV pancreatic cancer [41].

An experimental study by Zhong et al. studied the role of combining high-intensity focused ultrasound (HIFU) with an intra-arterial infusion of gemcitabine (IAG) in patients with intermediate and advanced pancreatic cancer. A total of 48 patients were included in the study, out of which 24 received HIFU with IAG and 24 were only treated with HIFU (control group). Compared to those in the control group, the overall response rate, level of pain relief, and one-year survival rate were higher in the group that received HIFU and IAG (p<0.05). This showed that combining HIFU with IAG can improve outcomes in patients with advanced pancreatic cancer [42].

In a phase II trial conducted by Heinrich et al., mitomycin C and gemcitabine-based IAC were administered into the celiac artery to understand the role of combining IAC and SC in patients with advanced pancreatic cancer. A total of 37 treatment cycles were performed on 17 patients. As per the radiographic and tumor marker criteria, four out of 17 and seven out of 17 patients showed an objective treatment response, respectively. The median progression-free and overall survivals were 4.6 and 9.1 months, respectively. The presence of distant metastases was significantly associated with the median survival, where those without distant metastases had a longer time of 15 months versus 7.1 months in those with distant metastases (p=0.037). It was concluded that IAC using mitomycin C and gemcitabine is more efficacious when compared to SC using gemcitabine and is almost comparable to the outcomes in the FOLFIRINOX (5-FU + oxaliplatin + irinotecan + leucovorin) regimen [43].

To understand the role of 24-hour continuous IAC with gemcitabine in patients with locally advanced pancreatic cancer, Beane et al. conducted the Regional Chemotherapy in Locally Advanced Pancreatic Cancer (RECLAP) study. This was performed after the patients underwent vascular redistribution of the inflow to the head of the pancreas by arterial coil embolization followed by perfusion catheter placement within the splenic artery. The median overall survival was 15.3 months, and the median time to progression was three months. Two patients had stable disease after four months and underwent pancreaticoduodenectomy. The primary pitfall of this study was the risk of gastrointestinal toxicity manifesting as duodenal ischemia and upper gastrointestinal bleeding. Hence, they concluded that 24-hour regimens have a higher incidence of adverse effects and, hence, shorter infusion schedules are more beneficial while administering IAC in locally advanced pancreatic cancer [44].

In an attempt to understand the implications for quality of life (QoL) after IAC and radiotherapy (IAC/RT) versus surgery alone in resectable pancreatic and periampullary cancers, Morak et al. performed a questionnaire-based study. The European Organization for Research and Treatment of Cancer QoL questionnaire C30 was utilized, and patients were advised to fill these out every three months during the first 24 months after randomization. Eighty-six patients completed one or more questionnaires, and a total of 355 could be used for assessment. The results showed that IAC/RT did not impair physical, emotional, or social functioning, and it was associated with less pain (p=0.02) and less nausea and vomiting (p=.0.01). Over 24 months, IAC/RT had better QoL improvement compared with observation alone in patients with resected pancreatic and periampullary cancer [45].

Biliary tract cancers

Edeline et al. performed a systematic review and pooled analysis of various locoregional therapies in patients with intrahepatic cholangiocarcinoma to compare the roles of radioembolization (RE), TACE, and HAI in chemotherapy. The pooled mean overall survival was 14.1 months for RE, 15.9 months for TACE, and 21.3 months for HAI. When analyzed together, RE, TACE, and HAI had a pooled mean weighted OS of 15.7 months and 25.2 months in patients treated in the first line along with systemic chemotherapy. They concluded that, although the evidence to recommend is low, HAI with concomitant systemic chemotherapy is an alternate treatment protocol for managing intrahepatic cholangiocarcinomas [46].

In another meta-analysis performed by Ma et al., they studied the role of adjuvant chemotherapy in resectable intrahepatic cholangiocarcinomas. Although they did not specifically analyze the outcomes of HAI chemotherapy, their pooled data included HAI, TACE, and intravenous chemotherapy infusions. Pooled analysis showed significant benefit in the adjuvant chemotherapy group (HR 0.66, 0.55-079, P <.001, I-square [I] = 20.8%). Using a gemcitabine-based regimen was associated with significantly improved overall survival (p<0.001) [47].

To study the role of intraluminal radiofrequency ablation (IRA) and HAI in advanced biliary tract cancers, Gou et al. conducted a multicentre, retrospective controlled study in 135 patients. Sixty-four of them underwent stent placement along with IRA and HAI, whereas 71 underwent only stent placement. The HAI chemotherapeutic regimen included oxaliplatin, leucovorin, and 5-FU. The median stent patency time was significantly longer in the combined group (8.2 months vs. 4.3 months; p<0.001). Overall survival was also significantly higher in the combination group (13.2 months vs. 8.5 months; p<0.001). This proved that including HAI alongside IRA improves stent patency and longevity rates in patients with advanced biliary tract cancers [48].

Bladder cancer

The role of IAC in bladder cancer has been an evolving area of interest, where multiple studies were performed with or without concomitant systemic or intravesical chemotherapy (IVeC). Chen et al. studied the role of IAC combined with IVeC by comparing it with patients who only received IVeC in T1 grade 3 transitional cell carcinoma (BTCC) after bladder-preserving surgery. Sixty patients with T1G3 BTCC were randomized, and 29 received IAC and IVeC (group A). Thirty-one patients were managed with IVeC alone (group B). The IAC regimen included epirubicin and cisplatin; the intravesical regimen was epirubicin. Group A had a 10.3% recurrence rate compared to a 45.2% recurrence rate in group B. A 0% progression rate was registered in Group A, whereas Group B reported a 22.6% recurrence rate. Both the recurrence and progression rates were significantly lower in Group A (p=0.004 and p=0.011, respectively). The median time to first recurrence in both groups was 15 and 6.5 months, respectively. The overall survival rates were 96.6% and 87.1% in Groups A and B, respectively. Hence, combining IAC with IVeC can prolong the time of recurrence and progression when compared to intravesical chemotherapy alone [49].

Another study by Sun et al. evaluated the benefit of administering a combination of IAC and IVeC after transurethral resection of high-risk, non-muscle-invasive bladder cancer (NMIBC). Two hundred and eighty-three patients were randomly assigned, and 141 patients received IAC+ IVeC (group A), and 142 received only IVeC. The IAC regimen consisted of cisplatin and epirubicin, and the IVeC regimen was epirubicin-based. Group A had a recurrence rate of 29.1%, and the same in Group B was 42.9%. The difference was significant (p=0.01). The progression rate of Group A was 15.6%, and that of Group B was 25.3%, with a significant difference (p=0.039). This showed again that IAC, with IVeC, has superior outcomes in reducing postoperative recurrences and prolonging the time to recurrence in bladder cancer [50].

To understand the role of IAC with IVeC in patients post-transurethral resection of bladder tumors (TURBT) in T1 grade 3 bladder cancer, Huang et al. performed a randomized trial where the outcomes in 203 patients diagnosed with NMIBC and underwent TURBT were studied. Group A (n=68) consisted of those who received IAC+ IVeC, and Group B (n=135) consisted of those who were managed with IVeC only. Both the IAC and IVeC regimens were epirubicin-based. Group A had a recurrence rate of 35.8%, whereas in Group B, it was 41.8%, and the difference was significant (p<0.05). The progression rate in Group A was 20.7%, and that in Group B was 23.5%, with a significant difference (p<0.05). This again proved that IAC+IVeC is a beneficial regimen for managing bladder cancers [51].

A meta-analysis by Ji et al. talked about the efficacy and adverse reactions of IAC in patients with bladder cancer. The analysis concluded that IAC+IVeC had a significantly longer recurrence-free survival rate (HR = 0.55, 95% CI = 0.40-0.76, I2 = 0%) and progression-free survival rate (HR = 0.59, 95% CI = 0.37-0.97, I2 = 0%) compared to patients who received only IVeC after TURBT in NMIBC patients. Preoperative IAC had no significant overall survival benefit over preoperative IVC in bladder cancer. Hence, it can be concluded that combining IAC with IVeC in NMIBC can have better outcomes when compared to administering only IVeC [52].

Head and neck cancers

Head and Neck Squamous Cell Carcinoma (HNSCC)

In a phase II study by Rao et al. to understand the role of cisplatin IAC and erlotinib with concomitant radiation in patients with locally advanced HNSCC, 21 patients were administered this regimen over a seven-week treatment period. The overall survival rate was 63%, and the relapse and persistent disease rate was 36.8%. This improved overall survival showed that IAC with cisplatin combined with erlotinib is beneficial in patients with locally advanced HNSCC [53]. Yu et al. studied the safety and efficacy of using accelerated fractionation radiotherapy and late intensification with two cisplatin IAC infusions in patients with locally advanced HNSCC. Out of the 10 patients recruited, two had stage III, one had stage IVa, and seven had stage IVb disease. Six patients survived disease-free at 39 months and 67 months [54].

The randomized radiation and concomitant high-dose intra-arterial or intravenous cisplatin (RADPLAT) trial for advanced HNSCC studied the role of IAC with cisplatin versus IVC with cisplatin and concomitant radiotherapy in both arms. The locoregional control and overall survival were not different between the treatment arms. Late dysphagia was worse in the IVC arm (p=0.014). This proved that IAC cisplatin has no added benefit compared to IVC cisplatin therapy, despite a higher concentration of the drug delivered directly to the tumor [55]. A five-year QoL in patients post-RADPLAT trial showed that there were no significant changes since the trial, but xerostomia improved in patients (p=0.004). Survivors have lower fatigue levels and better voice and swallowing, all of which have significantly improved since the previous QoL analysis one year after the trial [56].

Maxillary Sinus Cancer

Homma et al. investigated the safety and efficacy of super selective cisplatin IAC and concomitant radiotherapy for patients with locally advanced maxillary sinus cancer (MSC) under the RADPLAT-MSC trial [57]. Following this, Makino et al. performed a pathological evaluation of the outcomes of this trial. Nineteen patients were enrolled, out of whom five showed T3 disease and 14 had T4 disease. The five-year overall survival rate was 67.1%, and the five-year disease-specific survival rate was 81.9%. With a low adverse effect incidence, it can be concluded that RADPLAT is an effective procedure for locally advanced MSC that can be used to avoid highly invasive surgery [58].

Laryngeal Cancer

Furusuka et al. evaluated the role of superselective IAC with concurrent chemoradiation therapy for functional laryngeal preservation in advanced laryngeal squamous cell cancer. Sixty-four patients received two cycles of IAC with cisplatin and docetaxel, plus continuous IVC with 5-FU and concurrent chemoradiation. The overall five- and 10-year survival rates were 70.4% and 62.9%, respectively, in patients with T3 cancer, and the five- and 10-year survival rates were 50.4% and 44.1%, respectively, in patients with T4a cancers. The five- and 10-year laryngeal preservation rates were 92.5% and 87.4% in patients with T3 cancer and 48.6% and 35.6%, respectively, in patients with T4a cancer. This showed that IAC has a definite role in the management of advanced laryngeal squamous cell cancer with functional laryngeal preservation [59].

Similarly, Furusuka et al. also performed a study to understand the laryngeal preservation rates in advanced piriform sinus squamous cell carcinomas by using IAC with cisplatin, docetaxel, and IVC with 5-FU. The five-year survival rates per the ‘T’ classification were 96% in T3, 48.1% in T4a, and 16.7% in T4b. The five-year laryngeal preservation rates by ‘T’ classification were 92.4% in T3, 36.2% in T4a, and 16.7% in T4b. This showed that super-selective IAC showed better laryngeal preservation rates and high survival rates, especially in T3 [60].

Tongue Cancer

Takayama et al. conducted a study to understand the role of IAC with proton beam therapy (PBT) in patients with stage III-IVB tongue cancer. 33 patients were enrolled, and the median follow-up duration was 43 months. The IAC regimen used was based on cisplatin and sodium thiosulfate. The three-year overall survival was 87%, progression-free survival was 74.1%, and local control and regional control rates were 86.6% and 83.9%, respectively. This showed that PBT-IAC for advanced tongue cancer had efficacious outcomes and could be used as an alternative regimen [61].

Non-small cell lung cancer (NSCLC)

Yuan et al. performed a retrospective study to understand the role of IAC in 40 patients with stage III NSCLC. The IAC regimen consisted of gemcitabine and cisplatin. Response evaluation was done as per RECIST criteria, and 19/40 (47.5%) exhibited a response to therapy after the completion of the first three cycles. The median time-to-progression was five months, and the median life expectancy was nine months. Hence, it was concluded that IAC has the potential to shrink tumor size and improve outcomes in advanced NSCLC [62].

In a prospective trial done by Nakanishi et al., 25 patients with stage III or IV or recurrent NSCLC without distant metastases (M1b) who were not eligible for standard chemotherapy or chemoradiotherapy were treated with an IAC regimen of docetaxel and cisplatin. Twenty-four of the 25 patients were available for long-term evaluation. The overall response rate was 52%, with a complete response in one patient and a partial response in 12. The median progression-free survival and overall survival periods were 6.5 months and 17.5 months, respectively. The one- and two-year survival rates stood at 81% and 32%, respectively. If an adequate number of feeder arteries can be identified for therapy, it was concluded that IAC is a beneficial alternative for advanced NSCLC patients who cannot tolerate standard chemotherapy or chemoradiation [63].

Malignant brain tumors

Glioblastoma

Despite years of research, the five-year survival rate in glioblastoma patients is about 5%, hence pushing researchers to identify better alternative regimens. Burkhardt et al. studied the role of IAC with bevacizumab after blood-brain barrier disruption in patients with recurrent glioblastoma. As a part of this prospective study, 14 patients who failed the standard treatment with temozolomide and radiation therapy were given a single IAC dose of bevacizumab, followed by a standard IVC using bevacizumab. The median progression-free survival was 10 months. The median overall survival was estimated to be 8.8 months. They have concluded that patients who have never received bevacizumab might respond well to the IAC+IVC bevacizumab regimen, and it can improve survival rates [64].

To understand the role of repeated super selective IAC (SIAC) with bevacizumab after blood-brain barrier disruption in newly diagnosed glioblastoma cases, Patel et al. evaluated the response of 23 patients to SIAC with bevacizumab along with standard chemoradiation. The isocitrate dehydrogenase mutation was found in one out of 23 patients, and the O(6)-methylguanine-DNA-methyltransferase (MGMT) status was available for 11 out of 23 patients (three methylated, seven unmethylated, and one inconclusive). The median progression-free period was 11.5 months, and the six-month, 12-month, 24-month, and 60-month progression-free survival rates were 91.3%, 47.4%, 32.5%, and 5.4%, respectively. Median overall survival was 23.1 months, with 12, 24, and 36-month overall survival rates of 77.3%, 45.0%, and 32.1%, respectively [65]. Chen et al. concluded that perfusion guidance can be a great tool while administering super selective IAC in cases of malignant brain tumors, and since this technology has already been used for stroke, it has been time-tested and concluded to be safe and efficacious. This paved the way for the use of mesenchymal stem cells for the management of glioblastomas, as perfusion guidance can enable better feeder artery identification, enabling efficient delivery of the cells directly to the tumor [66].

Oligodendroglial Tumors

Guillaume et al. studied the role of IAC with blood-brain barrier disruption in patients with aggressive oligodendroglial tumors. 13 patients with temozolomide-refractory anaplastic oligodendroglioma (n=11) or oligoastrocytoma (n=2) underwent blood-brain barrier disruption and IAC with carboplatin and melphalan. Simultaneous IVC with etoposide was also administered. Two patients had a complete response, three had a partial response, five showed a stable disease, and three progressed. Median overall progression-free survival was 11 months. It was identified that patients who showed partial or complete responses demonstrated deletions of chromosomes 1p and 19q in the tumor cells. Of the five patients with stable disease, two demonstrated 1p and 19q deletions, and three showed a 19q deletion only. Hence, it can be concluded that IAC in treatment-refractory cases of advanced oligodendroglial tumors can be an effective alternative, especially in patients with 1p or 19q deletions [67].

Locoregional and systemic complications of IAC

In the realm of drug delivery systems, IAC is recognized for its ability to achieve heightened drug concentrations at the intended site. However, this approach is not without its drawbacks, as it can give rise to a diverse range of side effects that may manifest either locally or throughout the body. Table 1 summarizes the common complications encountered.

**Table 1:** List of locoregional and systemic complications of using intra-arterial chemotherapy HAI: hepatic arterial infusion

Indication	Locoregional Complications	Systemic Complications
Retinoblastoma	Transient lid edema, forehead hyperemia, loss of nasal lashes, complications at the access site, vitreous hemorrhage, phthisis, retinal or choroidal vascular occlusions [11]; cataract [12]; diffuse arteriolar sclerosis, hyperpigmentation of retinal pigment epithelium, partial retinal atrophy [13]; retinal detachment [16]	Grade 3 or 4 neutropenia, grade 3 or 4 thrombocytopenia, Fever [11], nausea, vomiting [16]
Colorectal cancer with liver metastases (HAI)	Liver enzymes and bilirubin elevation [21,23,29]	Grade 4 thrombocytopenia, grade 4 hypokalemia, nausea, vomiting, constipation, and diarrhea [17, 29]; neutropenia (>= grade 3) [20,21,23,29];
Advanced uveal melanoma with hepatic metastases (HAI)	Catheter complications, liver toxicity, and liver enzyme elevation [30]	Grade 3 or higher thrombocytopenia and neutropenia [30]
Hepatocellular carcinoma (HAI)	Catheter-related complications, liver ischemia, sepsis [31]; and liver enzyme elevation [32,33,34]; bilirubin elevation, and hepatic vascular injury [33,34]	Grade 3 diarrhea [31]; anemia, thrombocytopenia, leukopenia, fever, and fatigue [32,33,34]
Gastric cancer	Liver enzyme elevation, ulceration, and reflux [37]	Anemia, leukopenia, thrombocytopenia, nausea, vomiting, neurological toxicity, stomatitis, and diarrhea [37]
Cervical cancer	-	Thrombocytopenia, anemia, leukopenia, nausea, and vomiting [39,40]
Pancreatic cancer	Grade 3 or 4 duodenal ischemia, upper gastrointestinal bleeding [44]	Nausea, vomiting, hair loss, bone marrow suppression, thrombocytopenia, and neutropenia [41]
Biliary tract cancers	Liver enzyme and bilirubin elevation, pancreatitis, peritonitis, biliary infection, bile duct perforation, and catheter-related complications [48]	Thrombocytopenia, neutropenia, nausea, vomiting, and diarrhea [48]
Bladder cancer	Bladder irritation, mucositis, haematuria, and pain [52]	Nausea, vomiting, neutropenia, liver enzyme elevation, and creatinine elevation [52]
Head and neck cancers	Xerostomia, dysphagia, voice abnormalities, bleeding, and catheter-related complications [54]; mucositis, and dermatitis [61]	Grade 4 leukopenia, acute skin reactions, thrombocytopenia, and fatigue [54]
Non-small cell lung cancer	Cough and chest pain [62]	Anorexia, pain, thrombocytopenia, fatigue [62]; and appetite loss [63]
Malignant brain tumors	Seizure, headache, and neurological symptoms [64–67]	Thrombocytopenia, neutropenia, fatigue, nausea, and vomiting [64–67]

## Conclusions

The field of IAC has made significant advancements since its initial development. The intervention has demonstrated notable enhancements in patient outcomes across various malignancies, particularly through the conversion of tumors that were previously deemed unresectable to resectable, as well as through its synergistic effects when combined with systemic chemotherapy or radiotherapy, resulting in improved patient outcomes. Numerous trials and studies have been conducted to examine the effectiveness of IAC, yielding inconsistent outcomes. Consequently, further clinical trials are warranted to elucidate the potential of IAC as a standard alternative or primary treatment regimen. Cancers such as retinoblastoma have been thoroughly investigated in terms of the effectiveness of IAC and have subsequently been integrated into established protocols for disease management. As outlined in the article, certain studies have demonstrated more favorable results with IAC compared to other conventional treatment methods. However, further trials are necessary to determine the statistical significance of the observed differences in outcomes. As advancements in vascular techniques continue to progress, there is a concurrent advancement in the development of newer and more effective methods for delivering IAC. This progress is paving the way for the evolution of future delivery systems.
